# Position in Reading

**Published:** 1876-07

**Authors:** 


It is best for the light to fall on the page from behind, a little to one side, the person sitting in an erect position.

All persons under parental control should be peremptorily forbidden to read by artificial light, except occasionally for half an hour at a time. This injunction would require no repetition, if parents could know as physicians do, in how many cases the sight is prematurely impaired, or actual disease of the eye is engendered, which, after heavy expense, and weeks and months of starvation and dreary confinement to darkened rooms, is found to be intractable.

It ought to be known that reading by gas-light is very much more injurious to the eyes than candle-light, from the flicker caused by the unsteady jet of the gas from its fountain, and also from the particular tinge of gas-light. A candle flickers some, this is remedied by having two candles burning at the same time; they should be rather behind the person, the eyes should never be allowed to face artificial light in reading.

The habit of reading by artificial light in bed, is so reprehensible, if for no other reason than by its perilling the lives of others by burning the house up, as has been the case in multitudes of instances, that it is not worth while to address any argument to those who practice it,—whose absorbing, predominant characteristics are recklessness and selfishness.

Many read in the daytime, while reclining on a sofa, or in bed, this occasions an unnatural strain of the sight, which may very well induce organic disease, by a persistence in the abnormal tension.

